# Incidence rate and clinical characteristics of acute endophthalmitis following 23-gauge pars plana vitrectomy

**DOI:** 10.1186/s40942-022-00435-8

**Published:** 2022-12-21

**Authors:** SeyedehMaryam Hosseini, Ghazale Daraee, Nasser Shoeibi, Elham Bakhtiari, Mohammad-reza Ansari-astaneh, Mojtaba Abrishami, Mehrdad Motamed Shariati

**Affiliations:** Eye research center, Mashhad University of Medical Sciences, Khatam Al-Anbia eye hospital, Gharani boulevard, Mashhad, Iran

**Keywords:** Pars plana vitrectomy, Surgical instruments, Acute postoperative endophthalmitis, Infectious endophthalmitis

## Abstract

**Purpose:**

In this study, we evaluated the incidence and clinical characteristics of post-vitrectomy acute endophthalmitis in a tertiary eye center.

**Methods:**

Data were obtained by reviewing the patients’ medical records who underwent primary pars plana vitrectomy (PPvitx) from September 2011 to March 2017. We excluded patients who had any ocular surgery in the past 6 months, immunocompromised patients, and patients with a pre-operative diagnosis of endophthalmitis. The primary outcome was the incidence of acute post-pars plana vitrectomy endophthalmitis.

**Results:**

Out of 6474 cases who underwent PPvitx, 12 cases of endophthalmitis (incidence rate of 0.18%) were identified. We found two positive cultures for staphylococcus epidermidis and one positive culture for staphylococcus aureus. Underlying causes of primary vitrectomy in patients who got endophthalmitis were diabetic retinopathy (8 cases), rhegmatogenous retinal detachment (2 cases), and the epiretinal membrane (1 case), and non-clearing vitreous hemorrhage secondary to central retinal vein occlusion (1 case).

**Conclusion:**

In the present study, the rate of post-vitrectomy acute endophthalmitis was higher than in other reported studies.

## Background

Infectious endophthalmitis is a potentially devastating and vision-threatening inflammation caused by an infectious agent involving intraocular tissues [1]. Most cases of infectious endophthalmitis are exogenous, resulting from the incubation of the organism from the external environment, which is due to trauma, eye surgery, or the spread of keratitis [2]. Cases of post-cataract surgery acute endophthalmitis are much more common than acute endophthalmitis following pars plana vitrectomy (PPvitx), which is very rare. The incidence of acute endophthalmitis following cataract surgery and intravitreal injections is 0.4% and 0.06%, respectively [3]. Acute post-vitrectomy endophthalmitis (APVE) is usually diagnosed within the first week, notably on the third day, after surgery, with a sign of intense intraocular inflammation [4]. It is unknown whether microincision vitrectomy surgery (MIVS) is a risk factor for endophthalmitis [5]. Preliminary studies have suggested that the nature of sutureless wound healing in MIVS predisposes the eye to endophthalmitis [5, 6]. However, early studies of a prospective nature noted ambiguous results, and recently published studies did not find a significant difference in the incidence of endophthalmitis in the two methods of 20-G and MIVS. Chen et al., in a systematic review and meta-analysis study, showed that the incidence of post-vitrectomy endophthalmitis is 0.04%, 0.03%, and 0.11% after 20-, 23-, and 25-gauge pars plana vitrectomy respectively. They concluded that there is no difference between the rate of endophthalmitis after 23-gauge and 20-gauge vitrectomy [5].

In this study, we investigate the incidence, clinical characteristics, and treatment outcomes of post-vitrectomy acute endophthalmitis for 6 years in a tertiary eye center in northeast Iran. As a developing country, due to economic issues, we have to use recycled single-use vitrectomy tools. A comparison of the results of this study with similar studies in developed countries can indicate the possible effect of tool reuse on the incidence of endophthalmitis.

## Methods

### Study design

The present retrospective study was conducted from September 2011 to March 2017 at Khatam-Al-Anbia tertiary eye Hospital, affiliated with Mashhad University of Medical sciences. Data were obtained by reviewing the patients’ medical records. This study followed the Declaration of Helsinki, and the Mashhad University of Medical Sciences ethical committee approved this study (IRB number: IR.MUMS.MEDICAL.REC.1396.966). The inclusion criterion was patients who underwent primary pars plana vitrectomy (PPvitx) with or without combined cataract surgery. We excluded patients who had any ocular surgery in the past 6 months, immunocompromised patients, and patients with a pre-operative diagnosis of endophthalmitis. Post-traumatic patients were also excluded from the study. The diagnosis of acute endophthalmitis following PPvitx was confirmed clinically based on the presence of hypopyon and/or vitritis, by two independent vitreoretinal surgeons in the first 6 weeks after the primary surgery. Collected data include patient demographics, underlying disease and the reason for primary vitrectomy, surgical procedure details such as the utilization of intraocular tamponades, the incidence and clinical characteristics of acute infectious endophthalmitis following vitrectomy and the visual outcomes.

The primary outcome was the incidence of acute post-pars plana vitrectomy endophthalmitis. The secondary outcomes include the indications of the primary vitrectomy, details of the primary surgery (tamponade agent), best-corrected visual acuity (BCVA), and relative afferent pupillary defect (RAPD) before the incidence and after the management of endophthalmitis, intraocular pressure (IOP), lens status and the treatment methods which was used for the management of endophthalmitis.

After diagnosing acute endophthalmitis, depending on the surgeon’s opinion and the patient’s condition, rescue pars plana vitrectomy and,or intravitreal vancomycin (1 mg/0.1 cc) and ceftazidime (2.25 mg/0.1 cc) injection was done for the patient. For all patients, systemic antibiotics (intravenous vancomycin 1 gr every 12 h + ceftazidime 1 gr every 8 h) were injected for 3 days.

### Surgical procedure

All patients underwent a 23-gauge 3-port pars plana vitrectomy. At the end of the surgery, subconjunctival cefazolin (50 mg) and betamethasone (2 mg) are injected. Scleral wounds are sutured with vicryl 8–0 if the wounds showed leakage. Because of the economic issues in our country, we have reused disposable vitrectomy devices, including vitrectomy cassettes, vitreous cutters, trocar cannulas, intraocular forceps, endoilluminators and laser probes after employment of the standard sterilization methods [7]. These tools had not been used for vitrectomy in the case of endophthalmitis.

### Sterilization protocol

The recycled items including vitrectomy cassettes, trocar cannulas, vitreous cutters, endoilluminators, intraocular forceps, and laser probes, are discarded after being used in infected eyes or at the surgeon’s discretion. The steps of our sterilization protocol are as follows: (1) Removing bulk materials using an enzymatic detergent. For the vitreous cutter tubing, we passed the detergent through the lumen for one minute before the next step. (2) The use of an ultrasonic cleaner, which is set at 40 °C for 10 min, for all devices except for vitrectomy cassettes. The inside and outside of vitrectomy cassettes are cleaned with soap detergent and rinsed with soap-free sterile water. (3) All devices are dried and packed. (4) Sterilization by Steri-Vac™ sterilizers using 100% ethylene oxide (EO) as per standard protocol.

We validate the sterilization process with class V EO indicator strips and EO biological indicators in our center.

### Statistical analysis

We used the Statistical Package for Social Sciences (SPSS) software version 19 (IBM SPSS Statistics, IBM Corporation, Chicago, IL) for statistical analysis. We utilized descriptive analysis to assess the patients’ demographics, cause of primary vitrectomy, surgical procedure details, and the incidence of post-vitrectomy acute endophthalmitis. We used the Wilcoxon test to compare visual acuity before and after the incidence of endophthalmitis. A P-value < 0.05 was considered significant.

## Results

The patient’s medical records evaluation showed that 6474 cases underwent pars plana vitrectomy from September 2011 to March 2017 at Khatam-Al-Anbia tertiary eye Hospital of Mashhad (Table 1). A total of 12 patients with endophthalmitis had been diagnosed. So, the incidence of acute endophthalmitis after pars plana vitrectomy was 1.85 per thousand or 0.18%. Four patients (33.3%) were male, and eight patients (66.7%) were female. The mean age of patients with acute endophthalmitis was 54.08 ± 12.95 years. Based on the medical records, nine patients (75%) had diabetes, and 1 (8.3%) had hypertension. Underlying causes of primary vitrectomy in patients who got endophthalmitis were diabetic retinopathy (8 cases), rhegmatogenous retinal detachment (2 cases), and the epiretinal membrane (1 case), and non-clearing vitreous hemorrhage secondary to central retinal vein occlusion (1 case). In 5 patients of the endophthalmitis group, a tamponade agent was used (three cases with silicone oil and 2 cases with gas). Of all patients with endophthalmitis, 7 had intravitreal bevacizumab (Avastin^®^) injection, and only 1 had cataract surgery at the same time as primary vitrectomy. Trans-operative injection of bevacizumab was done at the end of the surgery from one of the sclerotomies. Bevacizumab was extracted from a new bottle with 29 G needle.

For six patients (50%), rescue vitrectomy was done with tamponade of silicone oil in 5 of them. For the others, intravitreal vancomycin (1 mg/0.1 cc) and ceftazidime (2.25 mg/0.1 cc) were injected.

Clinical characteristics of patients with post-vitrectomy endophthalmitis were summarized in Table 2. All cases of endophthalmitis after vitrectomy occurred in the first week after the surgery.

Visual acuity of the patients was in the range of 3/10 to hand motion (HM) after the first surgery and before the incidence of post-vitrectomy endophthalmitis. In 6 patients (50%), visual acuity was counting fingers (CF) and in 2 patients (16.7%) was hand motion with projection. We used the logMAR scale to analyze the visual data quantitatively. In this way, we considered 1.85 logMAR as the vision of counting fingers, 2.30 as the vision of hand motion, 2.48 as the vision of light perception, and three as the vision of no light perception (NLP) [8]. The lens status was phakic in 9 patients (75%) and pseudophakic in 3 (25%) patients at endophthalmitis presentation. The relative afferent pupillary defect (RAPD) was positive in the affected eye of ten patients. The RAPD of the other two patients was not assessable. The mean ± SD IOP was 13.33 ± 2.57mmHg.

After endophthalmitis treatment, in the last follow-up visit (with an average of 1 year), patients’ visual acuity varied between counting fingers and NLP, with 5 cases of hand motion, 3 cases of light perception, 1 case of finger count, and 3 cases of NLP. The visual acuity of patients after vitrectomy before the incidence of endophthalmitis compared to the visual acuity after the treatment of endophthalmitis is summarized in Table 3. As we showed, visual acuity had been significantly reduced (P = 0.002). Lens status was phakic in 8 and pseudophakic in 4 patients after endophthalmitis treatment.

Regarding the causative organism for endophthalmitis, we found two positive cultures for staphylococcus epidermidis and one positive culture for staphylococcus aureus. The other cases were culture-negative.

**Table 1:** Clinical characteristics of patients who underwent pars plana vitrectomy between 2011–2017

Age (mean ± SD)	Sex		Cause of primary vitrectomy				Concurrent cataract surgery		Tamponading agents		
	Male	Female	Diabetic retinopathy	RRD	Macular surgery	Others	Yes	No	Silicone oil	Gas	No
53.9 ± 13.01	3056 (47.2)	3418 (52.8)	2577 (39.8)	1580 (24.4)	1483 (22.9)	834 (12.9)	3872 (59.8)	2602 (40.2)	1936 (29.9)	920 (14.2)	3618 (55.9)

**Table 2:** Clinical characteristics of patients with endophthalmitis

Patient no.	Gender	Cause of the primary vitrectomy	Concurrent procedure	Using a tamponading agent in the primary PPvitx	Treatment method
1	Male	Diabetic retinopathy	Intravitreal bevacizumab	–	Rescue vitrectomy + intravitreal antibiotics
2	Female	Diabetic retinopathy	Intravitreal bevacizumab	–	Intravitreal antibiotics
3	Female	Diabetic retinopathy	Intravitreal bevacizumab	SF6 20%	Intravitreal antibiotics
4	Male	Rhegmatogenous retinal detachment	–	Silicon oil	Rescue vitrectomy + intravitreal antibiotics
5	Male	Rhegmatogenous retinal detachment	–	Silicon oil	Rescue vitrectomy + intravitreal antibiotics
6	Female	Epiretinal membrane	Cataract surgery	–	Rescue vitrectomy + intravitreal antibiotics
7	Female	Diabetic retinopathy	Intravitreal bevacizumab	–	Intravitreal antibiotics
8	Female	Non-clearing vitreous hemorrhage secondary to CRVO	–	–	Rescue vitrectomy + intravitreal antibiotics
9	Female	Diabetic retinopathy	Intravitreal bevacizumab	Silicon oil	Intravitreal antibiotics
10	Male	Diabetic retinopathy	Intravitreal bevacizumab	–	Intravitreal antibiotics
11	Female	Diabetic retinopathy	Intravitreal bevacizumab	SF6 20%	Rescue vitrectomy + intravitreal antibiotics
12	Female	Diabetic retinopathy	–	–	Intravitreal antibiotics

**Table 3:** Visual status of patients with endophthalmitis

	Number	Minimum (logMAR)	Maximum (logMAR)	Mean (logMAR)	SD	Test statistics^a^	
Visual acuity after the first surgery	12	0.5	2.3	1.47	0.73	Z = − 3.071	P-value = 0.002
Visual acuity at the last follow-up visit (1 year) after the incidence of endophthalmitis	12	1.85	3	2.48	0.35		

^a^Wilcoxon test

## Discussion

This study investigated the incidence, clinical characteristics, and visual outcomes of acute post-vitrectomy endophthalmitis (APVE). According to the results of this study, out of 6474 cases of vitrectomy performed between 2011 and 2017 in Khatam Al-Anbia tertiary Ophthalmology Hospital, 12 cases of acute endophthalmitis occurred. The incidence of acute endophthalmitis during this period was 0.18%. We summarize several previous articles on the APVE incidence following 23-gauge PPvit in Table 4 [7, 9–12]. Previous studies have reported different rates of APVE incidence (0.02–0.14%). The condition of the wound at the end of the operation, the duration of surgery, the immune status of the patient, and the use of tampons such as silicone oil or gas at the end of the surgery are factors that can explain this difference [13, 14]. Numerous studies have been performed to evaluate the effect of sutureless minimally invasive vitrectomy surgery (MIVS) on the incidence of APVE, and the results are controversial [14]. While the results of some studies indicate a higher incidence of APVE following MIVS compared to the 20-gauge method, a recent meta-analysis showed that the incidence of APVE following 23-gauge PPvit (0.03%) was not significantly different from the 20-gauge method (0.05%) [15, 16]. However, this meta-analysis also showed a significantly higher incidence of APVE in patients operated on with the 25-gauge method [15]. The incidence of APVE in this study seems to be higher than in similar cases. Surgery in our patients was performed with the 23-gauge system, and the sclerotomies were sutured in all cases with wound leakage. Furthermore, hypotonia was not seen in any of the eyes after the first surgery. Various factors may explain the high incidence of APVE in this study. Due to the educational tasks of this center, several surgeries are performed by retinal fellowship assistants which may lead to a longer duration of the surgeries. However, due to the lack of evaluation of the duration of surgeries in our patients, it was not possible to test this hypothesis. While most APVE patients in this study had diabetes (66.7%), about 40% of all patients underwent PPvit for diabetic retinopathy. Immune system dysfunction might be a risk factor for infection in diabetic patients [17]. Higher APVE rates in diabetics have not been proven in previous studies [18]. The point to consider in this study was that in 3 patients (25%) with APVE, silicone oil was used after the first surgery. The incidence of APVE is infrequent in SO-filled eyes [5, 19]. However, it is essential to note that in this study, we did not evaluate the role of tamponade agents in preventing APVE. We live in a developing country and have to reuse some surgical equipment due to economic issues. This may be a sort of explanation for a little bit higher incidence of endophthalmitis in this center, although we use standard sterilization techniques [20–24]. In addition, an application of 10% povidone-iodine, wound suturing, and postoperative subconjunctival and topical antibiotics are recommended measures to reduce the incidence of endophthalmitis [6, 12, 25–27], which have been done for the patients of this study. In a 13 year retrospective study, Sukhum et al. reported 13 cases of endophthalmitis over a total of 12,989 pars plana vitrectomy operations. They concluded that endophthalmitis rates in those undergoing PPV using recycled single-use instruments were within the range of previously published results in which vitrectomy tools were disposed of after one use[22]. Regarding the similar standard sterilization method, the results were not compatible with ours.

The role of prophylactic antibiotics in preventing APVE is unclear. Different routes of using antibiotics at the end of the elective PPvit, including topical eye drops, local injections (subconjunctival, intravitreal, and intracameral), and systemic (oral) have been assessed, which indicates the lack of agreement in this regard [28–31]. In this study, in all participants, subconjunctival cefazolin (50 mg) and betamethasone (2 mg) were injected at the end of surgery.

Evaluation of the patient’s vision in this study indicates a significant decrease (P = 0.002) following the incidence of APVE. The mean ± SD of visual acuity was 2.48 ± 0.35 logMAR unit at the last follow-up visit (1 year after the incidence of APVE). This finding is consistent with the results of previous studies [12]. It seems that APVE is acute and occurs shortly after vitrectomy, which often means that despite immediate and aggressive treatment, visual results are poor. Several treatment options have been proposed for APVE. In milder cases, intravitreal injection of antibiotics can help control the infection. In more severe cases, rescue vitrectomy is inevitable [7, 12, 32]. In this study, 50% of cases underwent rescue vitrectomy with intravitreal antibiotics injection. In a similar study, Sukhum et al. showed a rate of 62% for rescue vitrectomy in patients with APVE. Given the advancement of PPvit techniques, it would be reasonable to lower the threshold for a rescue vitrectomy in the case of APVE.

In this study, three patients (25%) were culture-positive (two positive cultures for staphylococcus epidermidis and one positive culture for staphylococcus aureus). The rate of positive microbial culture was very variable in previous studies (0–100%) [10, 11]. However, the incidence of APVE is rare, and the sample size is small in similar studies. The most common APVE-producing organisms are gram-positive cocci [30]. Although rare cases of fungal endophthalmitis (Aspergillus flavus) have been reported following PPvit [7], these cases are usually limited to patients with immunodeficiency. So far, positive culture results for organisms including Staphylococcus lugdunensis, Proteus mirabilis, Aspergillus flavus, Staphylococcus epidermidis, Enterococcus faecalis, Staphylococcus aureus, methicillin-sensitive Staphylococcus aureus, coagulase-negative staphylococcus, methicillin-resistant Staphylococcus epidermis has been reported [5, 7, 10, 11, 33–35].

One of the limitations of this study is its retrospective nature of the study. Furthermore, the duration of surgery can be an influential factor that we have not been able to assess. Also, due to the small number of cases, it was impossible to assess the role of systemic diseases such as diabetes in the incidence of APVE. Besides, we did not have the data about how many times the devices were re-used.

It is recommended that the results of the present study be supplemented with other similar studies with larger sample sizes and more extended periods regarding the time of surgery.

**Table 4:** A review of previous studies regarding the incidence and clinical characteristics of APVE after 23-gauge PPvit compared to the present study

	Study time interval	Sample size (number of all vitrectomy surgeries)	The incidence rate of APVE (percent)	Mean final VA (LogMAR)	Positive culture of vitreous samples (%)
Present study	2011–2017	6474	0.18%	2.48	25%
Silpa‑archa et al. [7]	2005–2017	9102	0.09%	0.9	45%
Lin et al. [9]	2011–2014	3979	0.08%	1.75	67%
Xiang-yu et al. [11]	2002–2012	632	0	–	–
Lihteh et al. [12]	2005–2009	10,845	0.03%	2.02	67%
Oshima et al. [10]	2003–2008	6660	0.03%	–	100%

## Conclusion

The primary outcome of this study is the incidence rate of acute endophthalmitis following 23-gauge PPvitx with the reuse of surgical instruments (despite standard sterilization protocols), which was 0.18%. This rate is somehow higher than the estimated rate mentioned in previous studies.

## Data Availability

The data that support the findings of this study are available from the corresponding author upon reasonable request.

## Declarations

### Declarations

**Ethics approval and consent to participate**

This study is by the declaration of Helsinki, and it was approved by the institutional review board and ethics committee of the Mashhad University of Medical Sciences (approval number: IR.MUMS.MEDICAL.REC.1396.966).
